# Concurrent depression and anxiety in women undergoing additional diagnostic procedures due to positive screening for cervical cancer

**DOI:** 10.1371/journal.pone.0342492

**Published:** 2026-02-13

**Authors:** Irena Ilic, Goran Babic, Sandra Sipetic Grujicic, Ivana Zivanovic Macuzic, Milena Ilic, Ana Ravic-Nikolic, Vesna Milicic

**Affiliations:** 1 Faculty of Medicine, University of Belgrade, Belgrade, Serbia; 2 Department of Gynecology and Obstetrics, Faculty of Medical Sciences, University of Kragujevac, Kragujevac, Serbia; 3 Institute of Epidemiology, Faculty of Medicine, University of Belgrade, Belgrade, Serbia; 4 Department of Anatomy, Faculty of Medical Sciences, University of Kragujevac, Kragujevac, Serbia; 5 Department of Epidemiology, Faculty of Medical Sciences, University of Kragujevac, Kragujevac, Serbia; 6 Department of Dermatovenerology, Faculty of Medical Sciences, University of Kragujevac, Kragujevac, Serbia; University of Zagreb School of Medicine: Sveuciliste u Zagrebu Medicinski fakultet, CROATIA

## Abstract

**Introduction:**

Undergoing additional diagnostic procedures due to a positive cervical cancer screening in women can lead to psychosocial burden. This study investigated the co-occurrence of depression and anxiety in women both before and after additional diagnostic procedures for cervical cancer.

**Methods:**

This prospective observational study with repeated measures was conducted in a cohort consisting of all consecutive women (N = 172) who received an abnormal Pap screening test result and therefore presented to a gynecologist for additional diagnostic examinations. Multivariate logistic regression was used to identify the independent predictors of the concurrent depression and anxiety occurrence among women while undergoing additional diagnostic procedures due to positive screening for cervical cancer.

**Results:**

The prevalence of concurrent depression/anxiety among women while undergoing additional diagnostic procedures due to positive screening for cervical cancer was elevated from 34.3% before diagnostics to 46.5% after diagnostics (P < 0.05). Also, the prevalence of more severe concurrent depression/anxiety was higher after diagnostics than before diagnostics. Multivariate logistic regression presented that worry (regarding cervical cancer, general health, the result of the next cytology test, and having sex) was an independent significant predictive factor for concurrent depression/anxiety in women before undergoing additional diagnostic procedures due to positive screening for cervical cancer. Multivariate logistic regression presented that urban place of residence, history of induced abortion, and sedative use were predictive factors for concurrent depression and anxiety in women after undergoing additional diagnostic procedures due to positive screening for cervical cancer.

**Conclusion:**

Concurrent depression and anxiety occurrence was highly prevalent among women while undergoing additional diagnostic procedures due to positive screening for cervical cancer. This prospective observational study showed a correlation between certain risk factors and concurrent depression/anxiety, although it is difficult to determine causality of this association due to the limitations of the study design. Only evidence that establishes causality can definitively guide the implementation of specific procedures and interventions during cervical cancer screening aimed at reducing concurrent depression/anxiety.

## Introduction

Cervical cancer was the fourth most frequent cancer among women worldwide (6.9% of all), and ranked as the fourth leading cancer in mortality in women worldwide (8.1% of all) in 2022 [1]. Unlike many other malignancies, cervical cancer occurs at a slightly younger age, ranking as the second leading cancer in women aged 35–49 in the world in 2022 [1]. In Europe, cervical cancer ranked as the second leading cancer among women aged 20–39 in 2022 [1].

Over the last years in Serbia, a downward trend in the age-standardized rates of incidence and mortality of cervical cancer was observed [1,2], however the burden remains substantial. In 2022, the age-standardized incidence rate was 19.1 per 100,000, and the mortality rate was 5.9 per 100,000 [2]. Importantly, fewer than one third of cases in Serbia are discovered at an early stage of illness, with most cases detected in later stages [3]. About 2.8 million women aged ≥20 in Serbia are considered at risk for cervical cancer [2]. The World Health Organization’s Global Strategy to Accelerate the Elimination of Cervical Cancer, adopted in 2020, aims to achieve a threshold of 4 per 100,000 women-years for incidence by 2030, with 70% of women screened with a high-performance test by the ages 35 and 45 [4].

Organized population screening for cervical cancer was implemented in Serbia in 2013 [3,5]. The screening test for cervical cancer in Serbia is the cytological smear of the cervix (Pap test), with women who receive abnormal results referred for further diagnostics (consultative colposcopy, biopsy, endocervical curettage). These procedures must be performed no later than four to six weeks after referral from the health center. Having to undergo additional diagnostic procedures due to a positive cervical cancer screening presents a unique psychological stressor for women, as it introduces uncertainty regarding a potentially life-threatening diagnosis and such circumstances are recognized as triggers of distress and can lead to psychological and psychosocial burden in women [6–9].

A considerable number of women worldwide receive abnormal screening results and have to undergo further examinations such as colposcopy and biopsy. These diagnostic procedures are a burden for women because they have to deal with constant uncertainty and fear of developing cancer, creating negative emotions ranging from psychosexual distress, concerns about general health, fear for future offspring, dissatisfaction with sexual relations and quality of life, anxiety, depression, and fear of death [6,8,10,11]. Anxiety and depression are among the most commonly reported psychological reactions associated with cancer screening and follow-up diagnostic procedures [6,12]. These are distinct conditions: anxiety is characterized by immoderate worry, fear, and discomfort, while depression includes tenacious sadness, loss of interest, and feelings of hopelessness [13,14]. Nevertheless, these conditions can sometimes co-occur, as an emotional response to stressful situations, such as facing a difficult problem or an important decision that must be made [15–18]. Studies based on general and clinical samples showed that concurrent depressive/anxiety symptoms are common [19–21].

However, existing studies have focused on adverse effects among women undergoing cervical screening and occurrence of depression and anxiety [22,23] before the screening test or during the wait for the results of the screening test. Contrary to that, until this study [7], the available literature had no data about the occurrence of depression and anxiety in women with a positive Pap screening test during further diagnostic procedures. Therefore, this manuscript aimed to investigate the co-occurrence of depression and anxiety in women with a positive Pap screening test assessed both before and after additional diagnostic procedures within the organized cervical cancer screening program in Serbia.

## Methods

### Study setting and study population

This study was carried out in Kragujevac, a city in Central Serbia. In 2013, the Republic of Serbia started organized screening program for early detection of cervical cancer. In Serbia, the National Cancer-Screening Guidelines recommend the use of the Papanicolaou (Pap) test for cervical screening. In women aged 20–65 years who are invited to undergo cervical cancer screening every three years, Pap test is free for all women. For those women who had a positive result, additional diagnostic procedures are indicated (colposcopy/biopsy/endocervical curettage) that are to be attended in the next four to six weeks [5].

### Study design

This prospective observational study with repeated measures was conducted in a cohort of all consecutive women who received an abnormal Pap screening test result and therefore presented to a gynecologist for additional diagnostic examinations at the Clinic for Gynecology and Obstetrics of the Clinical Center.

### Study sample

The inclusion criteria include the following: (I) receiving a positive Pap test result and undergoing diagnostic procedures at the Gynecology and Obstetrics Clinic of the Clinical Center in Kragujevac; (II) age from 20 to 65 years; (III) fluency in speaking, reading and writing Serbian; (IV) able to independently fill out the surveys; (V) voluntary consent to participate in the study; (VI) and absence of exclusion criteria.

The exclusion criteria include: (I) with an age less than 20 years or older than 65; (II) previous cancer of the cervix or intervention on the cervix; (III) pregnancy that occurred during the study; (IV) presence of psychiatric diseases; (V) existence of diseases of the reproductive organs for which treatment was ongoing during the study; (VI) refusal to participate in the research; (VII) or presence of any other objective reason that prevents or hinders participation in the study.

## Sample size calculation

A research study that examined psychosocial state of women who received a positive cervical cancer screening result was used to inform the sample size calculation [24]. Calculation employed the Fleiss formula with continuity correction and study power for unmatched cohort studies and cross-sectional studies, assuming a two-tailed test, probability of making a type 1 error of 0.05, desired power of 95%, yielding a minimum necessary sample of 118. This was increased by 10% to compensate for potential errors in questionnaires’ filling out. The Epi Info (Version 7.2.0.1, Centers for Disease Control and Prevention, Atlanta, Georgia) was used to conduct sample size estimation.

### Data collection

Self-reported data were collected. Study participants completed a demographic-epidemiologic survey, together with the “Hospital Anxiety and Depression Scale, HADS” [25], “Cervical Dysplasia Distress Questionnaire, CDDQ” [24], and “Process and Outcome Specific Measure, POSM” [27].

**Fig 1:**
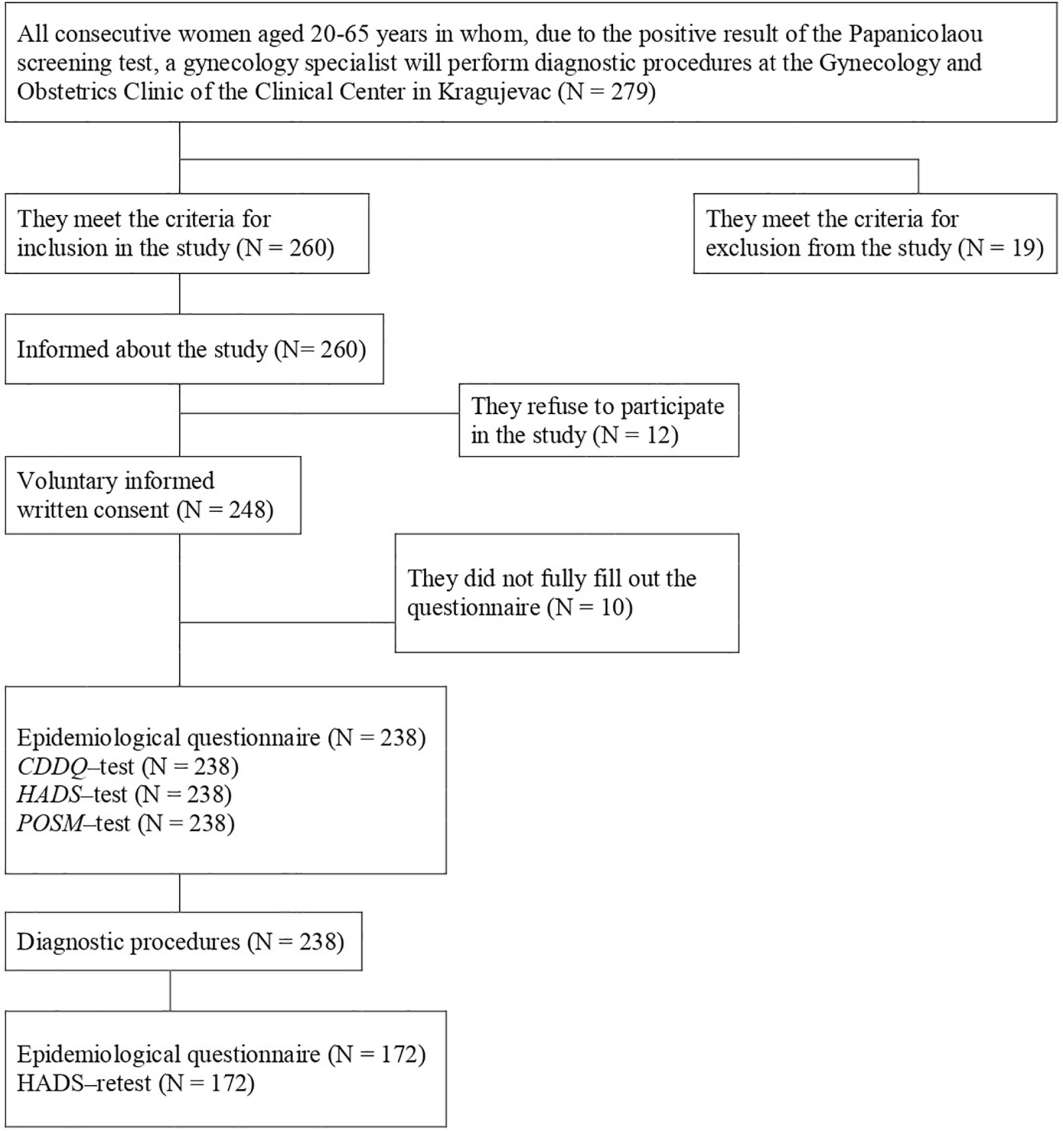
Research flow diagram. Abbreviations: CDDQ (Cervical Dysplasia Distress Questionnaire); HADS (Hospital Anxiety and Depression Scale); POSM (Process and Outcome Specific Measure).

In this study, participants were recruited between January 1 and December 31, 2017. In total, 260 eligible women were invited to participate in this study (Fig 1). The reasons for refusing to participate in the study (N = 12) were lack of time, lack of interest in the study, visual impairment and insufficient literacy. In addition, some women (N = 10) did not completely fill out the questionnaire. On recruitment, participants’ baseline characteristics were collected (N = 238). All enrolled participants underwent assessment of depression and anxiety using the HADS on the day of undergoing diagnostic procedures and on the day of receiving the results of the performed diagnostics (first: before performing the diagnostic procedures; and second: before receiving the results of the performed diagnostics). Only participants who responded to both the first and second surveys (N = 172) were included in this analysis. The response rate was 72.3%. The research was conducted in 2017.

### Instruments

The demographic-epidemiologic survey collected data about age, place of residence, educational level, occupation, marital status, body mass index, gynecological and reproductive characteristics (menarche, pregnancy, parity, menopause, use of oral contraceptives, abortion history), habits (tobacco smoking, use of alcohol), family history of cervical cancer, etc.

The primary outcome was cervical-cancer-screening-correlated concurrent depression and anxiety. Depression and anxiety were measured using the Hospital Anxiety Depression Scale (HADS) [25]. The HADS is a self-report scale of 14 items scored on a 4-point Likert scale (range 0–3). It is designed to measure anxiety (by the Hospital Anxiety and Depression Scale – Anxiety subscale, HADS-A) and depression (by the Hospital Anxiety and Depression Scale – Depression subscale, HADS-D), with 7 items for each subscale. The HADS is a screening tool that is used to estimate the degree of anxiety and depression, and for each subscale the score is the sum of the respective seven items (ranging from 0–21). Firstly, for the purposes of this study, respondents’ classification was dichotomized: an HADS subscale’s score ≥ 8 was considered indicative for anxiety and/or depression, and a score <8 was considered indicative of no anxiety/depression. Also, the severity of anxiety and depression was assessed based on the HADS-A and HADS-D score as mild anxiety/depression (score: 8–10), moderate anxiety/depression (score: 11–14) and severe anxiety/depression (score: 15–21) [28].

In this study, a respondent who had both HADS-A score ≥ 8 and HADS-D score ≥ 8 at the same time point of the study was considered a case of concurrent depression/anxiety. Respondents who did not have concurrent depression/anxiety were all other respondents who did not meet the specified criteria.

The Cervical Dysplasia Distress Questionnaire (CDDQ) is a specific scale for measuring psychological distress in women who received an abnormal Papanicolaou test result in the past year [26]. The CDDQ is a 23-item questionnaire that measures four domains of psychological distress characteristic to women testing positive on a cervical cancer screening test: two domains related to medical procedures (“Tension and discomfort” and “Embarrassment”) and two domains related to the consequences of receiving a positive result of the Papanicolaou test (“Concerns about sexual and reproductive consequences” and “Concerns about health consequences”).

The Process and Outcome Specific Measure (POSM) is a specific self-assessment scale for psychosocial burden of women undergoing screening for cervical cancer, and it was constructed within the TOMBOLA study [25]. Its questions refer to the period between receiving the abnormal screening result and completing the questionnaire. A higher score indicates greater psychosocial burden. The POSM questionnaire consists of 2 factors: factor 1 related to worry, and factor 2 related to satisfaction with information/support [29].

In this study, Serbian versions of all used scales (HADS, CDDQ and POSM questionnaires) were valid and reliable instruments for the assessment of psychological effects in women who are to undergo additional diagnostic procedures due to receiving a positive screening result for cervical cancer [30–32]. The Serbian version of the HADS demonstrated high internal consistency for both subscales (Cronbach’s alpha coefficient for subscale anxiety was 0.862, and for depression, 0.851), and the intra-class correlation coefficients for the two components were significant (0.860 and 0.843, p < 0.001) [30]. The factor analysis of the Serbian version of the CDDQ scale indicated four main components that showed good internal consistency (tension and discomfort = 0.844; embarrassment = 0.864; sexual and reproductive consequences = 0.867; and health consequences = 0.913), while the test–retest reliability coefficients were significant at the 0.01 level for all subscales [29]. Structure and reliability of the Serbian version of the POSM were confirmed: factor analysis demonstrated two factors (the worry factor, and the satisfaction with information/support factor), while the Cronbach’s alfa coefficients were 0.662 and 0.574, respectively [32].

### Statistical analysis

Descriptive and analytical statistical methods were used. Categorical variables were expressed as count (percentage), and continuous data were displayed as mean and standard deviation (SD). The independent variables were all sociodemographic and epidemiological characteristics of the participants. The outcome was concurrent depression and anxiety, both before and after undergoing diagnostic examinations. Comparisons of HADS-A and HADS-D score were determined by Student’s t-test, chi-square test and by Wilcoxon signed-rank test, as appropriate. For exploring the characteristics of participants that could be the associated factors of concurrent depression and anxiety, univariate and multivariate logistic regression were performed. Only variables (potentially associated factors) significant at the p < 0.05 level based on the univariate logistic regression models were included in the multivariate logistic models. Multivariate logistic regression was employed to determine the Odds Ratio (OR), with corresponding 95% confidence interval (95% CI) in order to estimate the independent association of concurrent depression and anxiety with participants’ characteristics. In order to correct for multiple comparisons, Bonferroni correction was used to adjust the significance level. This analysis used the `enter` method (default with the menu system). Models fit were assessed by the Hosmer-Lemeshow test of goodness of fit and Cox and Snell’s and Nagelkerke’s Pseudo R square measures. The model for concurrent depression and anxiety before diagnostics was statistically significant (Omnibus goodness of fit test: Chi-square = 26.86, df = 3, p < 0.001; Hosmer-Lemeshow goodness of fit test: Chi-square = 19.48, df = 8, p = 0.012). Cox and Snell’s and Nagelkerke’s Pseudo R square measures were 0.15 and 0.20, respectively. Among the observed variables, multicollinearity was examined with Variance Inflation Factors (VIF). The results show that all independent variables had a VIF near 1 (that is, VIF was from 1.01 to 1.07), indicating that there is no correlation between this independent variable and others. The model for concurrent depression and anxiety after diagnostics was statistically significant (Omnibus goodness of fit test: Chi-square = 34.49, df = 6, p < 0.001; Hosmer-Lemeshow goodness of fit test: Chi-square = 7.13, df = 8, p = 0.523). Cox and Snell’s and Nagelkerke’s Pseudo R square measures were 0.28 and 0.38, respectively. Among the observed variables, multicollinearity was examined with Variance Inflation Factors (VIF). The results show that all independent variables had a VIF near 1 (that is, VIF was from 1.02 to 1.09), indicating that there is no correlation between this independent variable and others. Statistical significance was considered at the p < 0.05 level. The SPSS Software (version 20.0, Chicago, IL, USA) was used to perform all statistical analyses.

### Ethical considerations

This study was approved by the Ethics Committee of the Faculty of Medical Sciences, University of Kragujevac (Ref. No.: 01–2176) and by the Ethics Committee of the Clinical Center Kragujevac (Ref. No.: 01–2869). All procedures were performed in accordance with the ethical standards of the institutional and/or national research committee and with the 1964 Helsinki declaration and its later amendments or comparable ethical standards. Informed, written, voluntary consent was obtained from all individual participants included in this study before participation in this study, and confidentiality was protected.

## Results

All consecutive women aged 20–65 years in whom, due to the positive result of the Papanicolaou screening test, a gynecology specialist will perform additional diagnostic procedures at the Gynecology and Obstetrics Clinic of the Clinical Center in Kragujevac (N = 279) were invited to participate in this study (Fig 1). Among them, 19 women were not eligible to participate in this study. A total of 260 women were eligible for participation in this study, but 22 of them were excluded because they refused to participate in the study (N = 12) or they did not fill out the questionnaires completely (N = 10). The remaining eligible 238 women were assessed with HADS, CDDQ and POSM scores before diagnostic procedures (this way, the basic value of the level of anxiety and depression in the time before the diagnostic procedures was determined), and were followed up to receipt of the results of the gynecological examination (this way, the psychological impact of the performed diagnostic procedures was determined). In this study, a repeat survey was conducted 2–4 weeks after the initial survey. During the follow-up, 66 women were excluded because of loss to follow-up, and the remaining 172 participants were included in the final analysis. The socio-demographic and other characteristics of the subjects with a positive Papanicolaou test result who participated in the first (Study time-point I) and second (Study time-point II) time points of the study did not differ significantly (Tables S1-13 in S1 File).

Mean score for anxiety in women undergoing cervical cancer screening according to the HADS scale for the HADS-A domain was 7.7 before diagnostic procedures, while after diagnostics it was an average of 8.2 (Table 1). Mean score for depression in women undergoing cervical cancer screening according to the HADS scale for the HADS-D was 5.6 before diagnostic procedures, while after diagnostics it was an average of 6.4.

**Table 1:** Descriptive statistics for anxiety and depression of study participants (N = 172), according to the Hospital Anxiety Depression Scale (HADS).

*Variables*	Mean ± SD (Range), Min-Max		
*HADS subscales*	Before	After	P*
HADS-Anxiety	7.7 ± 4.2 (0-21), 0-18	8.2 ± 4.3 (0-21), 0-18	0.286
HADS-Depression	5.6 ± 4.2 (0-21), 0-19	6.4 ± 4.1 (0-21), 0-20	0.086

SD (Standard Deviation). * Wilcoxon signed-rank test.

**Fig 2:**
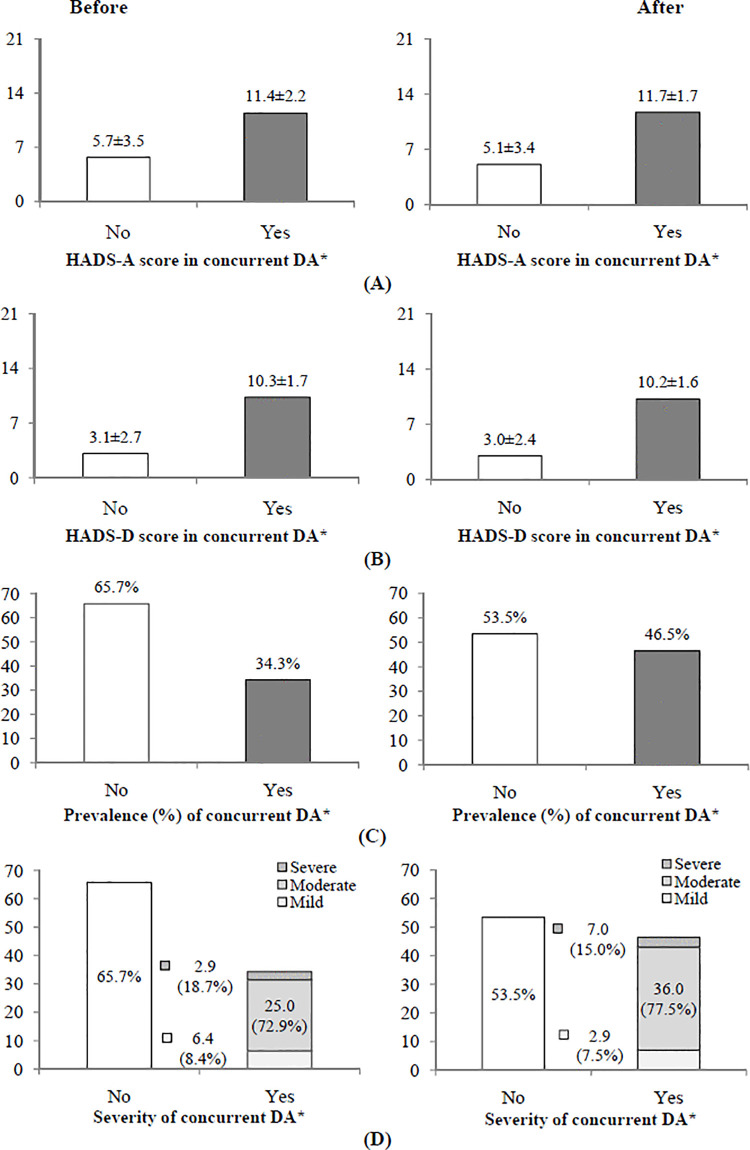
Comparison of depression/anxiety (DA) prevalence and severity among women with concurrent depression and anxiety while undergoing additional diagnostic procedures due to positive screening for cervical cancer. **(A)** HADS-A (Hospital Anxiety and Depression Scale – anxiety subscale) score in concurrent DA, **(B)** HADS-D HADS-D (Hospital Anxiety and Depression Scale – depression subscale) score, in concurrent DA, **(C)** prevalence of concurrent DA, **(D)** severity of concurrent DA. Comparison between two groups was determined by t-test, chi-square test or Wilcoxon signed-rank test. * (P < 0.05).

In women with concurrent depression and anxiety before undergoing additional diagnostic procedures (N = 59/172) due to positive screening for cervical cancer, the HADS-A score was higher (11.4 ± 2.2) compared with women without concurrent depression and anxiety (N = 133/172) before diagnostics (5.7 ± 3.5), P < 0.05 (Fig 2A). Also, in women with concurrent depression and anxiety after undergoing additional diagnostic procedures (N = 80/172) due to positive screening for cervical cancer, the HADS-A score was higher (11.7 ± 1.7) compared with women without concurrent depression and anxiety (N = 92/172) after diagnostics (5.1 ± 3.4), P < 0.05 (Fig 2A).

In women with concurrent depression and anxiety before undergoing additional diagnostic procedures (N = 59/172) due to positive screening for cervical cancer, the HADS-D score was higher (10.3 ± 1.7) compared with women without concurrent depression and anxiety (N = 133/172) before diagnostics (3.1 ± 2.7), P < 0.05 (Fig 2B).

Also, in women with concurrent depression and anxiety after undergoing additional diagnostic procedures (N = 80/172) due to positive screening for cervical cancer, the HADS-D score was higher (10.2 ± 1.6) compared with women without concurrent depression and anxiety (N = 92/172) after diagnostics (3.0 ± 2.4), P < 0.05 (Fig 2B).

The prevalence of concurrent depression/anxiety among women while undergoing additional diagnostic procedures due to positive screening for cervical cancer was elevated from 34.3% before diagnostics to 46.5% after diagnostics (P < 0.05) (Fig 2C).

Comparison of severity for concurrent depression/anxiety among women while undergoing additional diagnostic procedures due to positive screening for cervical cancer showed that prevalence of more severe concurrent depression/anxiety was higher after diagnostics than before diagnostics, as consequence of higher frequency of moderate concurrent depression/anxiety (36.0% versus 25.0%, respectively; P < 0.05) (Fig 2D).

Women with concurrent depression and anxiety before undergoing additional diagnostic procedures due to positive screening for cervical cancer more often were older, did not have children, were postmenopausal, more often used sedatives, more frequently had concerns about health consequences, and more often had worries (regarding cervical cancer, general health, the result of the next cytology test, and having sex), while they less often were laborers by occupation in comparison to women without concurrent depression/anxiety (Table 2).

**Table 2:** Descriptive statistics of women with concurrent depression and anxiety (DA) while undergoing additional diagnostic procedures due to positive screening for cervical cancer.

	Concurrent DA	
	Before diagnostics	After diagnostics
Variables	Number (%)	
Age (≥ 50 years)	31 (52.5)*	35 (43.8)
Place of residence (Urban)	40 (67.8)	44 (55.0)**
Occupation (Laborer)	27 (45.8)*	31 (38.8)
Education level (> 8 years)	44 (74.6)	63 (78.8)
Marital status (With partner)	48 (81.4)	66 (82.5)
Regularity of menstruation (Yes)	42 (71.2)	55 (68.8)
Postmenopausal (Yes)	30 (50.8)**	36 (45.0)
Duration of menopause (≥ 6 years)	15 (50.0)	15 (41.7)*
Pregnancy (Ever)	54 (91.5)	73 (91.2)
Abortion history (Ever)	35 (64.8)	56 (76.7)
Spontaneous abortion (Ever)	13 (37.1)	23 (41.1)
Induced abortion (Ever)	30 (85.7)	45 (80.4)**
Children (Yes)	54 (91.5)*	71 (88.8)
Number of sexual partners (≥ 3)	17 (34.0)	25 (34.2)
Oral contraceptive use (Ever)	7 (11.9)	9 (11.2)
Tobacco use (Ever)	32 (54.2)	48 (60.0)
Alcohol use (Ever)	8 (13.6)	12 (15.0)
Body Mass Index (≥ 25 kg/m^2^)	25 (42.4)	32 (40.0)
Sedative use (Yes)	27 (45.8)**	33 (41.2)**
Family history of cervical cancer (Yes)	8 (13.6)	10 (12.5)
Family history of non-cervical gynecological cancers Yes)	9 (15.3)	12 (12.5)
Family history of non-gynecological cancers (Yes)	14 (23.7)	23 (28.7)
Personal history of depression (Yes)	7 (11.9)	8 (8.0)
Personal history of anxiety (Yes)	1 (1.7)	0 (0.0)
Psychological distress by CDDQ subscales (Mean ± SD)		
Tension and discomfort	1.9 ± 0.6	2.0 ± 0.6**
Embarrassment	2.0 ± 0.9	2.0 ± 0.9
Concern about sexual and reproductive consequences	1.6 ± 0.5	1.6 ± 0.6
Concern about health consequences	2.7 ± 0.9*	2.7 ± 0.8*
Psychosocial burden by POSM subscales (Mean ± SD)		
Worry	48.7 ± 10.4**	45.9 ± 12.4**
Satisfaction with information/support	44.4 ± 13.9	42.9 ± 14.8**

CDDQ: the Cervical Dysplasia Distress Questionnaire; POSM: Process and Outcome Specific Measure; SD: Standard Deviation. * Probability (P < 0.05) by χ^2^-test, ** Probability (P < 0.05) by t-test.

Women with concurrent depression and anxiety after undergoing additional diagnostic procedures due to positive screening for cervical cancer less often had an urban place of residence, history of induced abortion, duration of menopause ≥ 6 years, while they more often used sedatives, often had psychosocial distress (tension and discomfort, concern about health consequences), they were more likely to have worries (regarding cervical cancer, general health, the result of the next cytology test, and having sex), and they were rarely satisfied with information/support in comparison to women without concurrent depression/anxiety.

Univariate logistic regression displayed that postmenopausal status (OR = 2.04, 95%CI = 1.07–3.88, P = 0.029), sedative use (OR = 2.56, 95%CI = 1.32–4.99, P = 0.006), and worry regarding cervical cancer, general health, the result of the next cytology test, and having sex (OR = 1.06, 95%CI = 1.03–1.10, P < 0.001) were potential risk factors for concurrent depression and anxiety in women before undergoing additional diagnostic procedures due to positive screening for cervical cancer (Table 3). Multivariate logistic regression presented that worry (regarding cervical cancer, general health, the result of the next cytology test, and having sex) was an independent significant predictive factor for concurrent depression and anxiety in women before undergoing additional diagnostic procedures due to positive screening for cervical cancer (OR = 1.06, 95%CI = 1.03–1.10, P < 0.001).

**Table 3:** Predictors of concurrent depression and anxiety (DA) in women while undergoing additional diagnostic procedures due to positive screening for cervical cancer – before diagnostic procedures.

	Univariate logistic regression			Multivariate logistic regression		
Variables	OR	95% CI	P	OR	95% CI	P
Age (≥ 50 years)	1.80	0.95-3.41	0.070			
Place of residence (Urban)	0.66	0.33-1.33	0.244			
Occupation (Laborer)	0.81	0.64-1.03	0.092			
Education level (> 8 years)	0.71	0.34-1.50	0.368			
Marital status (With partner)	1.06	0.47-2.36	0.896			
Regularity of menstruation (Yes)	1.12	0.56-2.29	0.752			
Postmenopausal (Yes)	2.04	1.07-3.88	0.029*			
Duration of menopause (≥ 6 years)	0.81	0.31-2.11	0.667			
Pregnancy (Ever)	0.52	0.19-1.50	0.227			
Abortion history (Ever)	0.58	0.28-1.20	0.144			
Spontaneous abortion (Ever)	1.01	0.44-2.32	0.987			
Induced abortion (Ever)	0.88	0.26-2.74	0.777			
Children (Yes)	0.38	0.14-1.07	0.067			
Number of sexual partners (≥ 3)	0.80	0.55-1.16	0.238			
Oral contraceptive use (Ever)	0.63	0.25-1.58	0.321			
Tobacco use (Ever)	0.84	0.45-1.59	0.600			
Alcohol use (Ever)	0.65	0.27-1.56	0.335			
Body Mass Index (≥ 25 kg/m^2^)	1.20	0.63-2.27	0.582			
Sedative use (Yes)	2.56	1.32-4.99	0.006*			
Family history of cervical cancer (Yes)	1.81	0.66-4.98	0.248			
Family history of other gynecological cancers	2.08	0.78-5.56	0.144			
Family history of non-gynecological cancers	0.66	0.32-1.37	0.267			
Personal history of depression (Yes)	1.77	0.61-5.14	0.296			
Personal history of anxiety (Yes)	1.93	0.12-31.44	0.644			
Psychological distress by CDDQ subscales						
Tension and discomfort	1.06	0.63-1.78	0.826			
Embarrassment	1.13	0.81-1.59	0.476			
Sexual and reproductive consequences	1.41	0.86-2.38	0.174			
Health consequences	1.42	0.98-2.06	0.061			
Psychosocial burden by POSM subscales						
Worry	1.06	1.03-1.10	<0.001^*,**^	1.06	1.03-1.10	<0.001^*,**^
Satisfaction with information/support	0.98	0.96-1.01	0.393			

CDDQ: the Cervical Dysplasia Distress Questionnaire; POSM: Process and Outcome Specific Measure; SD: Standard Deviation; OR: Odds Ratio; 95% CI: 95% Confidence Interval; P: Probability. * Nominally significant (P < 0.05); ** Characteristic that remains significant after Bonferroni correction for multiple comparisons.

Univariate logistic regression displayed that urban place of residence (OR = 0.15, 95%CI = 0.07–0.33, P < 0.001), history of induced abortion (OR = 0.25, 95%CI = 0.07–0.96, P = 0.043), sedative use (OR = 2.23, 95%CI = 1.16–4.30, P = 0.016), tension and discomfort regarding colposcopy (OR = 1.72, 95%CI = 1.04–2.87, P = 0.036), worry regarding cervical cancer, general health, the result of the next cytology test, and having sex (OR = 1.03, 95%CI = 1.01–1.06, P = 0.019), and satisfaction with information/support (OR = 0.97, 95%CI = 0.95–0.99, P = 0.021) were potential determinants of concurrent depression and anxiety in women after undergoing additional diagnostic procedures due to positive screening for cervical cancer (Table 4). Multivariate logistic regression presented that urban place of residence (OR = 0.11, 95%CI = 0.03–0.33, P < 0.001), history of induced abortion (OR = 0.18, 95%CI = 0.04–0.77, P = 0.021), and sedative use (OR = 2.90, 95%CI = 1.12–7.55, P = 0.029) were predictive factors for concurrent depression and anxiety in women after undergoing additional diagnostic procedures due to positive screening for cervical cancer. After the Bonferroni correction, history of induced abortion and sedative use were no longer significant predictors of concurrent depression and anxiety in women after undergoing additional diagnostic procedures due to positive screening for cervical cancer. An independent significant predictor for concurrent depression and anxiety in women after undergoing additional diagnostic procedures due to positive screening for cervical cancer was urban place of residence (P < 0.001).

**Table 4:** Predictors of concurrent depression and anxiety (DA) in women while undergoing additional diagnostic procedures due to positive screening for cervical cancer – after diagnostic procedures.

	Univariate logistic regression			Multivariate logistic regression		
Variables	OR	95% CI	P	OR	95% CI	P
Age (≥ 50 years)	1.06	0.58-1.94	0.858			
Place of residence (Urban)	0.15	0.07-0.33	<0.001^*,**^	0.11	0.03-0.33	<0.001^*,**^
Occupation (Laborer)	0.90	0.71-1.13	0.350			
Education level (> 8 years)	1.03	0.50-2.14	0.938			
Marital status (With partner)	1.23	0.57-2.64	0.601			
Regularity of menstruation (Yes)	1.45	0.74-2.84	0.283			
Postmenopausal (Yes)	1.53	0.83-2.84	0.173			
Duration of menopause (≥ 6 years)	0.37	0.14-1.00	0.051			
Pregnancy (Ever)	0.49	0.19-1.28	0.145			
Abortion history (Ever)	1.58	0.77-3.26	0.212			
Spontaneous abortion (Ever)	1.44	0.65-3.15	0.368			
Induced abortion (Ever)	0.25	0.07-0.96	0.043*	0.18	0.04-0.77	0.021*
Children (Yes)	0.52	0.22-1.24	0.139			
Number of sexual partners (≥ 3)	0.84	0.59-1.19	0.315			
Oral contraceptive use (Ever)	0.52	0.22-1.24	0.139			
Tobacco use (Ever)	1.26	0.69-2.31	0.455			
Alcohol use (Ever)	0.73	0.33-1.62	0.432			
Body Mass Index (≥ 25 kg/m^2^)	1.04	0.56-1.91	0.907			
Sedative use (Yes)	2.23	1.16-4.30	0.016*	2.90	1.12-7.55	0.029*
Family history of cervical cancer (Yes)	1.74	0.63-4.79	0.288			
Family history of other gynecological cancers	1.50	0.56-4.01	0.419			
Family history of non-gynecological cancers	0.97	0.50-1.88	0.931			
Personal history of depression (Yes)	1.35	0.47-3.90	0.580			
Personal history of anxiety (Yes)	–	–	–			
Psychological distress by CDDQ subscales						
Tension and discomfort	1.72	1.04-2.87	0.036*			
Embarrassment	1.13	0.82-1.56	0.461			
Sexual and reproductive consequences	1.40	0.85-2.29	0.186			
Health consequences	1.39	0.98-1.97	0.067			
Psychosocial burden by POSM subscales						
Worry	1.03	1.01-1.06	0.019*			
Satisfaction with information/support	0.97	0.95-0.99	0.021*			

CDDQ: the Cervical Dysplasia Distress Questionnaire; POSM: Process and Outcome Specific Measure; SD: Standard Deviation; OR: Odds Ratio; 95% CI: 95% Confidence Interval; P: Probability. * Nominally significant (P < 0.05); ** Characteristic that remains significant after Bonferroni correction for multiple comparisons.

## Discussion

This study identified several predictors of concurrent depression and anxiety in women while undergoing additional diagnostic procedures (colposcopy/biopsy/endocervical curettage) due to positive screening for cervical cancer. The independent predictor of concurrent depression and anxiety before diagnostic procedures was worry by POSM subscale, while independent predictors after diagnostic procedures were rural place of residence, history of induced abortion and sedative use.

It is well-known that diagnostic procedures that women undergo after a positive Pap smear are associated with numerous adverse psychological and psychosocial consequences [33–35]. Therefore, it is surprising that there has been only one study that examined factors associated with the development of these outcomes, primarily with anxiety and/or depression [7].

This study demonstrated that concurrent depression and anxiety in women who had received an abnormal Pap screening test was present in about one third of women before further diagnostic procedures, and nearly half of women after diagnostic procedures. Another study in Serbia, although it did not examine depression, found that almost a quarter of women with an abnormal Pap test result obtained at the primary care centers requiring colposcopy and/or HPV testing in the hospital who received positive HPV testing results were significantly more anxious before the test than those who were HPV negative, while after receiving the test results there was no difference [36]. In the year prior to the abnormal Pap test, in women receiving care in six Boston-area community health centers in 2004–2005, 15.8% of women had depression, and among them 29.7% showed concurrent anxiety [37]. Although little is known about the comorbid depressive and anxiety symptoms among the general population of Serbia, the 2019 National Health Survey reported that 2.8% of the female population had current depressive symptoms according to the Patient Health Questionnaire, while a higher percentage (5.2%) of respondents with depression was obtained based on the self-reported prevalence of depression [38]. A population-based study among 55,885 women aged 30–79 years in Southwest China reported comorbid depressive and anxiety symptoms in 3.5%, whereby participants having experienced one and/or more stressful life events had strong positive associations with comorbid depressive and anxiety symptoms, with a dose-response relationship (P < 0.05) [39]. The occurrence of concurrent depression/anxiety identified using HADS in the present study cannot be interpreted mechanistically. However, prior literature has proposed several hypotheses that may suggest a complex context of factors, particularly involving hormonal fluctuations, exposure to adverse life events and stress, and genetic predisposition, while at the neurobiological level suggested plausible pathophysiological mechanisms include originating in the amygdala [40], genetic risk factors [41], serotonin signaling pathways – i.e., the serotonin 4 receptor [42], neuronal surface autoantibodies [43] and a high prevalence of these disorders in autoimmune diseases [44]. Importantly, these mechanisms have been proposed in the literature but were not examined in the current study and should be interpreted as hypotheses that warrant direct investigation in future research.

This study showed that worry (i.e., psychosocial burden regarding cervical cancer, general health, having sex, and the result of the next cytology test) was a predictor of concurrent depression and anxiety before diagnostic procedures. Women were most likely to be distressed by their abnormal smear result suggesting that women were aware of the meaning of their smear result. Also, it may be that the information that women received (or sourced for themselves) and/or the way in which the information was communicated, failed to provide adequate reassurance. Nevertheless, since there were no differences in satisfaction with information/support in participants with and without concurrent depression/anxiety before diagnostic procedures, it may be that women do not fully understand the information they receive about their smear result. Stressful life events as a risk factor for depressive and anxiety symptoms have been reported in previous studies [45,46], while possible interpretations of the mechanism of effect include chronic activation of the hypothalamic-pituitary-adrenal axis [47,48], oestradiol and progesterone influence [49,50], alterations of neuroimmune function [51], change in mitochondrial DNA and telomere length [52,53], etc. These biological responses may amplify psychological sensitivity to diagnostic uncertainty and medical interventions; however, these possible explanations require confirmation in future research. In this study, comparison of severity for concurrent depression/anxiety among women while undergoing additional diagnostic procedures due to positive screening for cervical cancer showed that prevalence of more severe concurrent depression/anxiety was higher after than before diagnostics, as a consequence of higher frequency of moderate concurrent depression/anxiety. Many studies have shown that more severe concurrent depression/anxiety is associated with poorer treatment success, poorer quality of life, lower adherence to treatment, increased risk of suicide, and higher treatment costs [54,55]. These possible consequences of observed findings suggest that health professionals in primary health care settings should provide appropriate psychological support to women enrolled in cancer screening in order to adequately manage symptoms of depression and anxiety.

This study found that women with rural residence had a higher risk of concurrent depressive and anxiety symptoms after diagnostic gynecological procedures. Significant differences in comorbid depressive and anxiety symptoms between rural and urban participants could primarily be explained by differences in environment [56,57]. The use of preventive care among rural women faces significant socioeconomic barriers, including limited access to health resources (lack of transportation, limited availability of services, difficulty navigating complex health systems, health insurance), financial constraints (costs of health care, limited financial resources, unemployment or underemployment), and social determinants (poverty, lack of education, social isolation), which affect this population and may hinder access to preventive care and engagement in preventive health habits, including the use of preventive services such as cancer screening and health education programs [58,59]. In addition, the process of accelerating urbanization and lifestyle changes that characterize Serbia and other developing countries, increase the access to information and preventive health measures decreasing the odds of undiagnosed depression and/or anxiety [60]. While findings of this study align with the biopsychosocial models of mental health, the here discussed factors provide a conceptual framework for understanding the observed association. By addressing these socioeconomic complexities, health systems can work to ensure that women in rural and urban areas have equal access to preventive care and the opportunity to achieve optimal health and well-being.

In this study, women with a history of induced abortion showed a lower risk of concurrent depressive and anxiety symptoms following diagnostic gynecological procedures. The results of studies on the correlation between induced abortion and depression and anxiety are contradictory, and the interpretation of the results of these studies is complex due to methodological issues and a multitude of potential confounding factors (involving previous or ongoing psychiatric illness, genetic or medical indications, lack of social support, poverty, responsibilities) [61] and as such should be done cautiously. Although some authors reported about induced abortion being a stressful adverse life event that can potentially cause emotional consequences (more sense of guilt, irritability, shame, self-judgment, and guilty feelings) [62,63], some other authors reported that there is no evidence that abortion harms women’s self-esteem or life satisfaction [64]. The inverse association of the history of induced abortion with concurrent depression/anxiety in this study could be attributed to the circumstances that women with induced abortion may have been in better health or that, when undergoing abortion, they had the opportunity for health professionals to give them advice and support for adherence to the cervical cancer screening program and further diagnostic procedures.

Sedative use was associated with concurrent depression/anxiety experienced both before and after diagnostic procedures. In the context of abnormal results of cervical screening, further diagnostics can trigger or exacerbate concurrent depression/anxiety. In this study, women who used sedatives showed a three-fold higher risk of concurrent depressive/anxiety symptoms after diagnostic gynecological procedures. Consistent with prior research [65], it could be hypothesized that perhaps the association between sedative use and concurrent depression/anxiety was specific to women who had more worries (regarding cervical cancer, general health, having sex, and the result of the next cytology test) or women who needed help to relax during procedures such as colposcopy, especially if they experienced tension and discomfort.

Overall, the findings of this study emphasize the importance of integrating psychological screening and timely psychosocial support into cervical cancer prevention programs. Tailored interventions, particularly for women with pronounced worry, rural place of residence, history of induced abortion and sedative use could help mitigate the burden of potential consequences of diagnostic procedures. Finally, this could enhance adherence to diagnostic pathways and improve overall health outcomes.

### Strengths and limitations of the study

According to the available literature, this is the first study specifically focused on identifying factors that lead to anxiety and depression in women with a positive Pap smear before and after undergoing diagnostic procedures. This research was conducted at a single center, thereby providing a sample without selection bias. Participants were recruited from the entire screening population, indicating that all women undergoing cervical cancer screening had an equal chance to participate in this research. Consequently, the study sample can be considered representative of the entire cervical cancer screening population in this setting. Also, only validated questionnaires were used in this study.

Nonetheless, this study has several limitations. In addition to the inherent constraints of the applied study design, the use of self-report questionnaires is a limitation. Although the privacy of all information is guaranteed, some respondents might have been reluctant to disclose symptoms of depression and/or anxiety so the possibility of information bias cannot be entirely ruled out. However, anxiety and depression in here presented participants were assessed using standardized, validated questionnaires, which minimize possible biases. The question regarding the representativeness of the sample always exists, either due to barriers to recruiting participants for the study or due to fears of confidentiality breaches. While study’s inclusion and exclusion criteria were based on research question and ensured contextual relevance, they may limit generalizability to women outside organized screening frameworks or to different healthcare systems. Further on, even though the response rate was 72.3% and comparable to those reported in similar surveys, the possibility of non-response bias cannot be excluded. However, since the differences in characteristics of participants in the first and second time-point of this study were not statistically significant, the implications of attrition rate on results of this study were unlikely. Also, the study did not determine the level of anxiety and depression prior to the Pap smear screening test, which could help to further clarify the impact of gynecological procedures on anxiety and depression. There was no clinical confirmation of anxiety and depression, nor was there any insight into their medical records. Also, this study did not provide data on other potential predictors of anxiety and depression (i.e., socioeconomic status, concerns about HPV infection, sexually transmitted disease status of sexual partners, etc.). Additionally, potential influence of other factors to which the participants may have been exposed in the meantime, that might affect the level of anxiety and depression before and after diagnostic procedures, cannot be fully excluded.

## Conclusions

In this study, concurrent depression and anxiety occurrence was highly prevalent among women while undergoing additional diagnostic procedures due to positive Pap screening for cervical cancer. Besides, the prevalence of concurrent depression and anxiety among women while undergoing additional diagnostic procedures was elevated from before the diagnostics to after diagnostics. Worry, history of induced abortion, use of sedatives and rural place of residence of women were identified as the independent determinants of concurrent depression/anxiety. In addition, the severity of concurrent depression/anxiety was higher after than before diagnostics, as a consequence of higher frequency of moderate concurrent depression/anxiety.

The findings of this study could support healthcare professionals in making decisions about the timely delivery of psychological support to women with a positive screening test for cervical cancer, with the aim of maximizing participation of women in diagnostic follow-ups thus enabling timely initiation of treatment and thereby reducing the number of complications and deaths.

## Supporting information

S1 File Supplementary tables. This file contains: Table S1: The socio-demographic characteristics of the subjects in the study. Table S2: The generative characteristics of the subjects in the study. Table S3: The reproductive characteristics of the subjects in the study. Table S4: The characteristics of sexual behavior of the subjects in the study. Table S5: The smoking status of the subjects in the study. Table S6: Distribution of respondents by alcohol consumption habits. Table S7: Distribution of respondents according to level of nutrition, physical activity and use of tranquilizers. Table S8: Family health history of the respondents. Table S9: Personal health history of respondents. Table S10: Anxiety and depression in the personal health history of the respondents. Table S11: Consequences of the screening procedure (Pap smear/colposcopy) in the subjects. Table S12: Psychological distress in cervical dysplasia according to the CDDQ scale. Table S13: Psychosocial status of respondents in cervical cancer screening according to the POSM scale.
(DOCX)
