# Supplementary material for: Concurrent depression and anxiety in women undergoing additional diagnostic procedures due to positive screening for cervical cancer
Source: PLoS One. 2026 Feb 13;21(2):e0342492. doi: 10.1371/journal.pone.0342492 (PMC12904401; doi:10.1371/journal.pone.0342492)
Supplement: S1 File — This file contains: Table S1: The socio-demographic characteristics of the subjects in the study. Table S2: The generative characteristics of the subjects in the study. Table S3: The reproductive characteristics of the subjects in the study. Table S4: The characteristics of sexual behavior of the subjects in the study. Table S5: The smoking status of the subjects in the study. Table S6: Distribution of respondents by alcohol consumption habits. Table S7: Distribution of respondents according to level of nutrition, physical activity and use of tranquilizers. Table S8: Family health history of the respondents. Table S9: Personal health history of respondents. Table S10: Anxiety and depression in the personal health history of the respondents. Table S11: Consequences of the screening procedure (Pap smear/colposcopy) in the subjects. Table S12: Psychological distress in cervical dysplasia according to the CDDQ scale. Table S13: Psychosocial status of respondents in cervical cancer screening according to the POSM scale. (DOCX) [file pone.0342492.s001.docx]

The socio-demographic characteristics of the subjects with a positive Papanicolaou test result who participated in the first (*Study time-point I*) and second (*Study time-point II*) time points of the study did not differ significantly (Table 1).

| **Table 1. The socio-demographic characteristics of the subjects in the study** | | | | | | | | | | | | | | |
| --- | --- | --- | --- | --- | --- | --- | --- | --- | --- | --- | --- | --- | --- | --- |
|  | ***Study time-point I***  ***(N=238)*** | |  | | ***Study time-point I&II***  ***(N=172)*** | | | | |  | | | | ***P*** |
| **Variables** | | **Number (%)** |  |  | |  | | **Number (%)** | | |  | |  | |
| ***Age (years)*** | |  |  |  | | |  | |  | | |  | | |
| - ≤30 | | 14 (5.9) |  |  | | | 12 (7.0) | |  | | |  | | |
| - 31-40 | | 67 (28.2) |  |  | | | 43 (25.0) | |  | | |  | | |
| - 41-50 | | 77 (32.4) |  |  | | | 42 (24.4) | |  | | |  | | |
| - 51-60 | | 56 (23.5) |  |  | | | 51 (29.7) | |  | | |  | | |
| - ≥61 | | 24 (10.1) |  |  | | | 24 (14.0) | |  | | | *0.245** | | |
| ***Place of residence*** | |  |  |  | | |  | |  | | |  | | |
| - Rural | | 46 (19.3) |  |  | | | 45 (26.2) | |  | | |  | | |
| - Urban | | 192 (80.7) |  |  | | | 127 (73.8) | |  | | | *0.117** | | |
| ***Place of birth*** | |  |  |  | | |  | |  | | |  | | |
| - Rural | | 40 (16.8) |  |  | | | 35 (20.3) | |  | | |  | | |
| - Urban | | 198 (83.2) |  |  | | | 137 (79.7) | |  | | | *0.368** | | |
| ***Occupation***** | |  |  |  | | |  | |  | | |  | | |
| - Housewife | | 50 (21.0) |  |  | | | 45 (26.2) | |  | | |  | | |
| - Farmer | | 4 (1.7) |  |  | | | 4 (2.3) | |  | | |  | | |
| - Laborer | | 90 (37.8) |  |  | | | 67 (39.0) | |  | | |  | | |
| - Clerk | | 58 (24.4) |  |  | | | 37 (21.5) | |  | | |  | | |
| - Expert | | 36 (15.1) |  |  | | | 19 (11.0) | |  | | | *0.094** | | |
| ***Education level*** | |  |  |  | | |  | |  | | |  | | |
| - Incomplete primary school | | 5 (2.1) |  |  | | | 5 (2.9) | |  | | |  | | |
| - Primary school | | 37 (15.5) |  |  | | | 32 (18.6) | |  | | |  | | |
| - Secondary school | | 144 (60.5) |  |  | | | 107 (62.2) | |  | | |  | | |
| - College | | 18 (7.6) |  |  | | | 9 (5.2) | |  | | |  | | |
| - Faculty | | 34 (14.3) |  |  | | | 19 (11.0) | |  | | | *0.142** | | |
| ***Marital status*** | |  |  |  | | |  | |  | | |  | | |
| - Single | | 13 (5.5) |  |  | | | 10 (5.8) | |  | | |  | | |
| - Married | | 195 (81.9) |  |  | | | 140 (81.4) | |  | | |  | | |
| - Divorced/Widow | | 30 (12.6) |  |  | | | 22 (12.8) | |  | | | *0.969** | | |
| ***Religious affiliation*** | |  |  |  | | |  | |  | | |  | | |
| - Orthodox | | 231 (97.1) |  |  | | | 168 (97.7) | |  | | |  | | |
| - Catholic | | 2 (0.8) |  |  | | | 0 (0.0) | |  | | |  | | |
| - Muslim | | 2 (0.8) |  |  | | | 2 (1.2) | |  | | |  | | |
| - Other | | 3 (1.3) |  |  | | | 2 (1.2) | |  | | | *0.900** | | |

*P (probability). ** χ^2^-test. ** For retirees, the occupation before retirement is entered.

The generative characteristics of the subjects with a positive Papanicolaou test result who participated in the first (*Study time-point I*) and second (*Study time-point II)* time points of the study did not differ significantly (Table 2).

| **Table 2. The generative characteristics of the subjects in the study** | | | | | | | | | | | | |
| --- | --- | --- | --- | --- | --- | --- | --- | --- | --- | --- | --- | --- |
|  | ***Study time-point I***  ***(N=238)*** | | |  | | ***Study time-point I&II***  ***(N=172)*** | | |  | | ***P*** |  |
| **Variables** | | **Number (%)** | |  |  | **Number (%)** | |  | |  | | |
| ***Menstruation – ever*** | |  |  | |  | |  |  | |  | | |
| - Yes | | 238 (100.0) |  | |  | | 172 (100.0) |  | |  | | |
| - No | | 0 (0.0) |  | |  | | 0 (0.0) |  | | *1.000** | | |
| ***Age of first menstruation*** | |  |  | |  | |  |  | |  | | |
| - ≤13 | | 135 (56.7) |  | |  | | 92 (53.5) |  | |  | | |
| - ≥14 | | 103 (43.3) |  | |  | | 80 (46.5) |  | | *0.546** | | |
| ***First menstruation – spontaneous*** | |  |  | |  | |  |  | |  | | |
| - Yes | | 222 (93.3) |  | |  | | 159 (92.4) |  | |  | | |
| - No | | 16 (6.7) |  | |  | | 13 (7.6) |  | | *0.745** | | |
| ***Menstruation - regularity*** | |  |  | |  | |  |  | |  | | |
| - Yes | | 169 (71.0) |  | |  | | 123 (71.5) |  | |  | | |
| - No | | 69 (29.0) |  | |  | | 49 (28.5) |  | | *0.912** | | |
| ***Menstruation – now*** | |  |  | |  | |  |  | |  | | |
| - Yes | | 154 (64.7) |  | |  | | 103 (59.9) |  | |  | | |
| - No | | 84 (35.3) |  | |  | | 69 (40.1) |  | | *0.352** | | |
| ***If there is no menstruation, why?*** | |  |  | |  | |  |  | |  | | |
| - Stopped spontaneously | | 80 (95.2) |  | |  | | 68 (98.6) |  | |  | | |
| - Stopped after gynecological surgery | | 2 (2.4) |  | |  | | 0 (0.0) |  | |  | | |
| - She recently gave birth | | 2 (2.4) |  | |  | | 1 (1.4) |  | | *0.460** | | |
| ***Menopause*** | |  |  | |  | |  |  | |  | | |
| - No | | 156 (65.5) |  | |  | | 104 (60.5) |  | |  | | |
| - Yes | | 82 (34.5) |  | |  | | 68 (39.5) |  | | *0.292** | | |
| ***Age of last menstruation***** | |  |  | |  | |  |  | |  | | |
| - ≤55 | | 75 (91.5) |  | |  | | 62 (91.2) |  | |  | | |
| - ≥56 | | 7 (8.5) |  | |  | | 6 (8.8) |  | | *0.951** | | |
| ***Duration of menopause***** | |  |  | |  | |  |  | |  | | |
| - ≤5 | | 42 (51.2) |  | |  | | 32 (47.1) |  | |  | | |
| - ≥6 | | 40 (48.8) |  | |  | | 36 (52.9) |  | | *0.613** | | |

*P (probability). ** χ^2^-test. ** For menopausal women.

The reproductive characteristics of the subjects with a positive Papanicolaou test result who participated in the first (*Study time-point I*) and second (*Study time-point II*) time points of the study did not differ significantly (Table 3).

| **Table 3. The reproductive characteristics of the subjects in the study** | | | | | | | | | |
| --- | --- | --- | --- | --- | --- | --- | --- | --- | --- |
|  | ***Study time-point I***  ***(N=238)*** | |  | ***Study time-point I&II***  ***(N=172)*** | |  | | ***P*** |  |
| **Variables** | | **Number (%)** |  | **Number (%)** | |  |  | |  |
| ***Pregnancy – ever*** | |  |  |  |  |  | |  |  |
| - Yes | | 216 (90.8) |  |  | 150 (87.2) |  | |  |  |
| - No | | 22 (9.2) |  |  | 22 (12.8) |  | | *0.253** |  |
| ***Age of first pregnancy***** | |  |  |  |  |  | |  |  |
| - ≤20 | | 58 (26.9) |  |  | 47 (31.3) |  | |  |  |
| - ≥21 | | 158 (73.1) |  |  | 103 (68.7) |  | | *0.352** |  |
| ***Total number of pregnancies*** | |  |  |  |  |  | |  |  |
| - 1 | | 25 (11.5) |  |  | 11 (7.3) |  | |  |  |
| - 2 | | 55 (25.5) |  |  | 33 (22.0) |  | |  |  |
| - ≥3 | | 136 (63.0) |  |  | 106 (70.7) |  | | *0.233** |  |
| ***Abortion*** | |  |  |  |  |  | |  |  |
| - No | | 76 (35.2) |  |  | 42 (28.0) |  | |  |  |
| - Yes | | 140 (64.8) |  |  | 108 (72.0) |  | | *0.149** |  |
| ***Total number of abortions*** | |  |  |  |  |  | |  |  |
| - 1 | | 46 (32.9) |  |  | 33 (30.5) |  | |  |  |
| - 2 | | 38 (27.1) |  |  | 30 (27.8) |  | |  |  |
| - ≥3 | | 56 (40.0) |  |  | 45 (41.7) |  | | *0.715** |  |
| ***Type of abortion*** | |  |  |  |  |  | |  |  |
| - Spontaneous | | 22 (15.7) |  |  | 14 (13.0) |  | |  |  |
| - Induced | | 86 (61.4) |  |  | 68 (63.0) |  | |  |  |
| - Both spontaneous and induced | | 32 (22.9) |  |  | 26 (24.0) |  | | *0.612** |  |
| ***Spontaneous abortion*** | |  |  |  |  |  | |  |  |
| - No | | 86 (61.4) |  |  | 68 (63.0) |  | |  |  |
| - Yes | | 54 (38.6) |  |  | 40 (37.0) |  | | *0.805** |  |
| ***Total number of spontaneous abortions*** | |  |  |  |  |  | |  |  |
| - 1 | | 42 (77.8) |  |  | 31 (77.5) |  | |  |  |
| - 2 | | 7 (13.0) |  |  | 4 (10.0) |  | |  |  |
| - ≥3 | | 5 (9.2) |  |  | 5 (12.5) |  | | *0.799** |  |
| ***Induced abortion*** | |  |  |  |  |  | |  |  |
| - No | | 22 (15.7) |  |  | 14 (13.0) |  | |  |  |
| - Yes | | 118 (84.3) |  | 94 (87.0) | |  | | *0.543** |  |
| ***Total number of induced abortions*** | |  |  |  |  |  | |  |  |
| - 1 | | 41 (34.7) |  |  | 33 (35.1) |  | |  |  |
| - 2 | | 35 (29.7) |  |  | 26 (27.7) |  | |  |  |
| - ≥3 | | 42 (35.6) |  |  | 35 (37.2) |  | | *0.944** |  |
| ***Children – ever*** | |  |  |  |  |  | |  |  |
| - Yes | | 210 (88.2) |  |  | 145 (84.3) |  | |  |  |
| - No | | 28 (11.8) |  |  | 27 (15.7) |  | | *0.249** |  |

*P (probability). ** χ^2^-test. ** For respondents who have ever been pregnant.

The characteristics of sexual behavior of subjects with a positive Papanicolaou test result who participated in the first (*Study time-point* I) and second (Stu*dy time-point II*) time points of the study did not differ significantly (Table 4).

| **Table 4. The characteristics of sexual behavior of the subjects in the study** | | | | | | | | | | | | | |
| --- | --- | --- | --- | --- | --- | --- | --- | --- | --- | --- | --- | --- | --- |
|  | ***Study time-point I***  ***(N=238)*** | |  | ***Study time-point I&II***  ***(N=172)*** | | | | |  | | ***P*** |  |  |
| **Variables** | | **Number (%)** |  | **Number (%)** | | | |  | |  | | | |
| ***First sexual intercourse – age***** | |  |  | |  |  | | |  | |  |  |  |
| - ≤20 | | 161 (74.9) |  | |  | 109 (73.2) | | |  | |  |  |  |
| - ≥21 | | 54 (25.1) |  | |  | 40 (26.8) | | |  | | *0.711** |  |  |
| ***Number of sexual partners***** | |  |  | |  |  | | |  | |  |  |  |
| - 1 | | 109 (50.7) |  | |  | 74 (49.6) | | |  | |  |  |  |
| - 2 | | 28 (13.0) |  | |  | 19 (12.8) | | |  | |  |  |  |
| - ≥3 | | 78 (36.3) |  | |  | 56 (37.6) | | |  | | *0.968** |  |  |
| ***Contraceptive pills*** | |  |  | |  |  | | |  | |  |  |  |
| - No | | 195 (81.9) |  | |  | 145 (84.3) | | |  | |  |  |  |
| - Yes | | 43 (18.1) |  | |  | 27 (15.7) | | |  | | *0.595** |  |  |
| ***Oral contraception – age****** | |  |  | |  |  | | |  | |  |  |  |
| - ≤25 | | 29 (67.4) |  | |  | 20 (74.1) | | |  | |  |  |  |
| - ≥26 | | 14 (32.6) |  | |  | 7 (25.9) | | |  | | *0.603** |  |  |
| ***Oral contraception – duration****** | |  |  | |  |  | | |  | |  |  |  |
| - <5 | | 38 (88.4) |  | 24 (88.9) | | |  | | | |  | |  |
| - ≥5 | | 5 (11.6) |  | |  | 3 (11.1) | | |  | | *0.948** |  |  |
| ***Oral contraception – interruptions****** | |  |  | |  |  | | |  | |  |  |  |
| - Yes | | 29 (67.4) |  | |  | 21 (77.8) | | |  | |  |  |  |
| - No | | 14 (32.6) |  | |  | 6 (22.2) | | |  | | *0.355** |  |  |

*P (probability). ** χ^2^-test. ** For age at first sexual intercourse and number of sexual partners. 88.8% of respondents provided information; **** * For subjects who used oral contraception.

The smoking status of the subjects with a positive Papanicolaou test result who participated in the first (*Study time-point I*) and second (*Study time-point II*) time points of the study did not differ significantly (Table 5).

| **Table 5. The smoking status of the subjects in the study** | | | | | | | | | | | |
| --- | --- | --- | --- | --- | --- | --- | --- | --- | --- | --- | --- |
|  | ***Study time-point I***  ***(N=238)*** | | |  | ***Study time-point I&II***  ***(N=172)*** | | |  | | ***P*** | |
| **Variables** | | **Number (%)** | |  | **Number (%)** | | |  | |  | |
| ***Cigarette smoking – ever*** | |  | |  |  |  | | |  | |  |
| - No | | 100 (42.0) | |  | 74 (43.0) | | | |  | |  |
| - Yes | | 138 (58.0) | |  | 98 (57.0) | | | |  | | *0.919** |
| ***Current smoking status*** | |  | |  |  |  | | |  | |  |
| - Non-smoker | | 100 (42.0) | |  | 74 (43.0) | | | |  | |  |
| - Former smoker | | 63 (26.5) | |  | 46 (26.7) | | | |  | |  |
| - Current smoker | | 75 (31.5) | |  | 52 (30.2) | | | |  | | *0.788** |
| ***Age of initiation of cigarette smoking***** | | |  |  |  |  | | |  | |  |
| - ≤20 | | 121 (87.7) | |  | 86 (87.8) | | | |  | |  |
| - ≥21 | | 17 (12.3) | |  | 12 (12.2) | | | |  | | *0.986** |
| ***Number of cigarettes smoked per day***** | |  | |  |  |  | | |  | |  |
| - ≤20 | | 132 (95.7) | |  | 94 (95.9) | | | |  | |  |
| - ≥21 | | 6 (4.3) | |  | 4 (4.1) | |  | | | *0.920** | |
| ***Quitting smoking cigarettes***** | |  | |  |  |  | | |  | |  |
| - Yes | | 63 (45.7) | |  | 46 (46.9) | | | |  | |  |
| - No | | 75 (54.3) | |  | 52 (53.1) | | | |  | | *0.845** |

*P (probability). ** χ^2^-test. ** For respondents who were ever smokers.

The alcohol consumption habits of female subjects with a positive Papanicolaou test result who participated in the first (*Study time-point I*) and second (*Study time-point II*) time points of the study did not differ significantly (Table 6).

| **Table 6. Distribution of respondents by alcohol consumption habits** | | | | | | | | | | | | | | |
| --- | --- | --- | --- | --- | --- | --- | --- | --- | --- | --- | --- | --- | --- | --- |
|  | ***Study time-point I***  ***(N=238)*** | | |  | ***Study time-point I&II***  ***(N=172)*** | | |  | | | ***P*** | |  |  |
| **Variables** | | **Number (%)** | |  | **Number (%)** | |  | | |  | | | | |
| ***Alcohol consumption*** | |  | |  | |  | | |  | | |  | |  |
| - No | | 187 (78.6) | |  |  | 142 (82.6) | | |  | | |  | |  |
| - Yes | | 51 (21.4) | |  |  | 30 (17.4) | | |  | | | *0.379* | |  |
| ***Frequency of consumption*** | |  | |  |  |  | | |  | | |  | |  |
| - Never | | 187 (78.6) | |  |  | 142 (82.6) | | |  | | |  | |  |
| - Every day | | 1 (0.4) | |  |  | 1 (0.6) | | |  | | |  | |  |
| - 1-2 times a week | | 7 (2.9) | |  |  | 5 (2.9) | | |  | | |  | |  |
| - 1-2 times a month | | 15 (6.3) | |  |  | 11 (6.4) | | |  | | |  | |  |
| - 1-2 times a year | | 28 (11.8) | |  |  | 13 (7.6) | | |  | | | *0.229* | |  |
| ***Average consumption amount (unit)*** | | |  |  |  |  | | |  | | |  | |  |
| - Never | | 187 (78.6) | |  |  | 142 (82.6) | | |  | | |  | |  |
| - 1 | | 36 (15.1) | |  |  | 20 (11.6) | | |  | | |  | |  |
| - ≥2 | | 15 (6.3) | |  |  | 10 (5.8) | | |  | | | *0.564* | |  |

*P (probability).* χ^2^-test.

There were no significant differences between the subjects with a positive Papanicolaou test who participated in the first (*Study time-point I*) and second (*Study time-point II*) time points of the study in terms of nutritional status, physical activity, and use of sedative medications (Table 7).

| **Table 7. Distribution of respondents according to level of nutrition, physical activity and use of tranquilizers** | | | | | | | | | | | | | | | |
| --- | --- | --- | --- | --- | --- | --- | --- | --- | --- | --- | --- | --- | --- | --- | --- |
|  | ***Study time-point I***  ***(N=238)*** | |  | | | ***Study time-point I&II***  ***(N=172)*** | | | |  | | ***P*** | |  |  |
| **Variables** | | **Number (%)** |  |  |  | **Number (%)** | | |  | | | |  | | |
| ***Body mass index (kg/m^2^)*** | |  | |  |  | |  | |  | |  | | |  |  |
| - ≤24.9 | | 144 (60.5) | |  |  | | 104 (60.5) | |  | |  | | |  |  |
| - ≥25.0 | | 94 (39.5) | |  |  | | 68 (39.5) | |  | | *0.994** | | |  |  |
| ***Playing sports*** | |  | |  |  | |  | |  | |  | | |  |  |
| - Yes | | 17 (7.1) | |  |  | | 13 (7.6) | |  | |  | | |  |  |
| - No | | 221 (92.9) | |  | | 159 (92.4) | |  | | | *0.874** | | | |  |
| ***Recreational activities*** | |  | |  |  | |  | |  | |  | | |  |  |
| - Yes | | 98 (41.2) | |  |  | | 67 (39.0) | |  | |  | | |  |  |
| - No | | 140 (58.8) | |  |  | | 105 (61.0) | |  | | *0.651** | | |  |  |
| ***Use of sedatives*** | |  | |  |  | |  | |  | |  | | |  |  |
| - No | | 166 (69.7) | |  |  | | 117 (68.0) | |  | |  | | |  |  |
| - Yes | | 72 (30.3) | |  |  | | 55 (32.0) | |  | | *0.710** | | |  |  |

*P (probability). ** χ^2^-test.

There was no significant difference between the subjects with a positive Papanicolaou test who participated in the first (*Study time-point I*) and second (*Study time-point II*) time points of the study in relation to family history of cervical cancer, other gynecological malignant tumors and malignant tumors of other localizations (Table 8).

| **Table 8. Family health history of the respondents** | | | | | | | | | | | | | | | | |
| --- | --- | --- | --- | --- | --- | --- | --- | --- | --- | --- | --- | --- | --- | --- | --- | --- |
|  | ***Study time-point I***  ***(N=238)*** | | | | |  | ***Study time-point I&II***  ***(N=172)*** | | | | | |  | ***P*** |  |  |
| **Variables** | | **Number (%)** | | | |  | **Number (%)** | | | | |  | |  | | |
| ***Cervical cancer*** | |  | | |  | |  | |  | |  | | |  | |  |
| - No | | 215 (90.3) | | |  | |  | | 155 (90.1) | |  | | |  | |  |
| - Yes | | 23 (9.7) | | |  | |  | | 17 (9.9) | |  | | | *0.941* | |  |
| ***Degree of kinship*** | |  | | |  | |  | |  | |  | | |  | |  |
| - First | | 15 (65.2) | | |  | |  | | 13 (76.5) | |  | | |  | |  |
| - Second | | 4 (17.4) | | |  | |  | | 0 (0.0) | |  | | |  | |  |
| - Third | | 3 (13.0) | | |  | |  | | 3 (17.6) | |  | | |  | |  |
| - Fourth | | 1 (4.3) | | |  | |  | | 1 (5.9) | |  | | | *0.904* | |  |
| ***Other gynecological malignant tumors*** | |  | | |  | |  | |  | |  | | |  | |  |
| - No | | 209 (87.8) | | |  | |  | | 154 (89.5) | |  | | |  | |  |
| - Yes | | 29 (12.2) | | |  | |  | | 18 (10.5) | |  | | | *0.590* | |  |
| ***Degree of kinship*** | |  | | |  | |  | |  | |  | | |  | |  |
| - First | | 22 (75.9) | | |  | |  | | 17 (94.4) | |  | | |  | |  |
| - Second | | 3 (10.3) | | |  | |  | | 0 (0.0) | |  | | |  | |  |
| - Third | | 3 (10.3) | | |  | |  | | 1 (5.6) | |  | | |  | |  |
| - Fourth | | 1 (3.4) | | |  | |  | | 0 (0.0) | |  | | | *0.200* | |  |
| ***Other localizations of malignant tumors*** | | | |  |  | |  | |  | |  | | |  | |  |
| - No | | 168 (70.6) | | |  | |  | | 124 (72.1) | |  | | |  | |  |
| - Yes | | 70 (29.4) | | |  | |  | | 48 (27.9) | |  | | | *0.740* | |  |
| ***Degree of kinship*** | |  | | |  | |  | |  | |  | | |  | |  |
| - First | | | 38 (54.3) | |  | |  | | 29 (60.4) | |  | | |  | |  |
| - Second | | | 23 (32.9) | |  | |  | | 11 (22.9) | |  | | |  | |  |
| - Third | | | 8 (11.4) | |  | |  | | 7 (14.6) | |  | | |  | |  |
| - Fourth | | | 1 (1.4) | |  | | |  | | 1 (2.1) |  | | | *0.687* |  |  |

*P (probability*: χ^2^-тест).

There were no significant differences between the subjects with a positive Papanicolaou test who participated in the first (*Study time-point I*) and second (*Study time-point II*) time points of the study in relation to personal health history for sexually transmitted diseases, tumors of other localizations and other chronic diseases (Table 9).

| **Table 9. Personal health history of respondents** | | | | | | | | | | | | | |
| --- | --- | --- | --- | --- | --- | --- | --- | --- | --- | --- | --- | --- | --- |
|  | | ***Study time-point I***  ***(N=238)*** |  | ***Study time-point I&II***  ***(N=172)*** | | |  | | | ***P*** | |  |  |
| **Variables** | **Number (%)** | |  | **Number (%)** | |  | | |  | | | | |
| ***Venereal disease*** |  | |  |  |  | | |  | | |  | |  |
| - No | 229 (96.2) | |  |  | 165 (95.9) | | |  | | |  | |  |
| - Yes | 9 (3.8) | |  |  | 7 (4.1) | | |  | | | *0.882* | |  |
| ***Type of sexually transmitted disease*** |  | |  |  |  | | |  | | |  | |  |
| - HPV infection | 6 (66.7) | |  |  | 5 (71.4) | | |  | | |  | |  |
| - Chlamydia infection | 0 (0.0) | |  |  | 0 (0.0) | | |  | | |  | |  |
| - Gonorrhea | 2 (22.2) | |  |  | 1 (14.3) | | |  | | |  | |  |
| - Syphilis | 0 (0.0) | |  |  | 0 (0.0) | | |  | | |  | |  |
| - Other | 1 (11.1) | |  |  | 1 (14.3) | | |  | | | *0.965* | |  |
| ***Cancer*** |  | |  |  |  | | |  | | |  | |  |
| - No | 224 (94.1) | |  |  | 161 (93.6) | | |  | | |  | |  |
| - Yes | 14 (5.9) | |  |  | 11 (6.4) | | |  | | | *0.831* | |  |
| ***Cancer – site*** |  | |  |  |  | | |  | | |  | |  |
| - Breast | 7 (50.0) | |  |  | 5 (45.5) | | |  | | |  | |  |
| - Thyroid gland | 3 (21.4) | |  |  | 3 (27.3) | | |  | | |  | |  |
| - Other | 4 (28.6) | |  |  | 3 (27.3) | | |  | | | *0.926* | |  |
| ***Other chronic diseases*** |  | |  |  |  | | |  | | |  | |  |
| - No | 191 (80.3) | |  |  | 138 (80.2) | | |  | | |  | |  |
| - Yes | 47 (19.7) | |  |  | 34 (19.8) | | |  | | | *0.996* | |  |
| ***Type of chronic disease*** |  | |  |  |  | | |  | | |  | |  |
| - Thyroid disease | 10 (22.7) | |  |  | 4 (11.8) | | |  | | |  | |  |
| - Heart and blood vessel disease | 16 (36.4) | |  |  | 16 (47.1) | | |  | | |  | |  |
| - Asthma | 9 (20.5) | |  |  | 7 (20.6) | | |  | | |  | |  |
| - Other | 9 (20.5) | |  |  | 7 (20.6) | | |  | | | *0.624* | |  |

*P (probability)*: χ^2^-test.

There were no significant differences in personal health history for anxiety and depression between the subjects with a positive Papanicolaou test who participated in the first (*Study time-point I*) and second (*Study time-point II*) time points of the study (Table 10).

| **Table 10. Anxiety and depression in the personal health history of the respondents** | | | | | | | | | | | | |
| --- | --- | --- | --- | --- | --- | --- | --- | --- | --- | --- | --- | --- |
|  | ***Study time-point I***  ***(N=238)*** |  | ***Study time-point I&II***  ***(N=172)*** | | |  | | | ***P*** | |  |  |
| **Variables** | **Number (%)** |  | **Number (%)** | | | |  |  | | | | |
| ***Anxiety*** |  |  |  |  |  | | | | |  | |  |
| - No | 234 (98.3) |  |  | 170 (98.8) |  | | | | |  | |  |
| - Yes | 4 (1.7) |  |  | 2 (1.2) |  | | | | | *0.989* | |  |
| ***Depression*** |  |  |  |  |  | | | | |  | |  |
| - No | 219 (92.0) |  |  | 157 (91.3) |  | | | | |  | |  |
| - Yes | 19 (8.0) |  |  | 15 (8.7) |  | | | | | *0.789* | |  |

*P (probability*: χ^2^-test).

There was no significant difference between the subjects with a positive Papanicolaou test who participated in the first (*Study time-point I*) and second (*Study time-point II*) time points of the study in relation to the consequences of the screening procedure (Papanicolaou test/colposcopy) (Table 11).

| **Table 11. Consequences of the screening procedure (Pap smear/colposcopy) in the subjects** | | | | | | | | | | | |
| --- | --- | --- | --- | --- | --- | --- | --- | --- | --- | --- | --- |
|  | ***Study time-point I***  ***(N=238)*** | |  | ***Study time-point I&II***  ***(N=172)*** | | | |  | | ***P*** |  |
| **Variables** | **Number (%)** | |  | **Number (%)** | | |  | |  | | |
| ***Pain*** |  | |  |  |  |  | | | |  |  |
| - No | 220 (92.4) | |  |  | 156 (90.7) |  | | | |  |  |
| - Yes | 18 (7.6) | |  |  | 16 (9.3) |  | | | | *0.529* |  |
| ***Pain – duration (days)*** |  | |  |  |  |  | | | |  |  |
| - Never | 220 (92.4) | |  |  | 156 (90.7) |  | | | |  |  |
| - 1-2 | 13 (5.5) | |  |  | 12 (7.0) |  | | | |  |  |
| - ≥3 | 5 (2.1) | |  |  | 4 (2.3) |  | | | | *0.597* |  |
| ***Bleeding*** |  | |  |  |  |  | | | |  |  |
| - No | 209 (87.8) | |  |  | 146 (84.9) |  | | | |  |  |
| - Yes | 29 (12.2) | |  |  | 26 (15.1) |  | | | | *0.463* |  |
| ***Bleeding- duration (days)*** |  | |  |  |  |  | | | |  |  |
| - Never | 209 (87.8) | |  |  | 146 (84.9) |  | | | |  |  |
| - 1-2 | 21 (8.8) | |  |  | 19 (11.0) |  | | | |  |  |
| - ≥3 | 8 (3.4) | |  |  | 8 (4.7) |  | | | | *0.310* |  |
| ***Increased vaginal discharge*** |  | |  |  |  |  | | | |  |  |
| - No | 187 (78.6) | |  |  | 138 (80.2) |  | | | |  |  |
| - Yes | 51 (21.4) | |  |  | 34 (19.8) |  | | | | *0.683* |  |
| ***Increased vaginal discharge- duration (days)*** | |  |  |  |  |  | | | |  |  |
| - Never | 187 (78.6) | |  |  | 138 (80.2) |  | | | |  |  |
| - 1-2 | 23 (9.7) | |  |  | 16 (9.3) |  | | | |  |  |
| - ≥3 | 28 (11.8) | |  |  | 17 (9.9) |  | | | | *0.533* |  |

*P (probability*: χ^2^-test).

There was no significant difference in psychological distress in cervical dysplasia between subjects with a positive Papanicolaou test who participated in the first (*Study time-point I*) and second (*Study time-point II*) time points of the study, according to the CDDQ scale (Table 12).

| **12. Psychological distress in cervical dysplasia according to the CDDQ scale** | | | | | | | | | | | | | |
| --- | --- | --- | --- | --- | --- | --- | --- | --- | --- | --- | --- | --- | --- |
|  | ***Study time-point I***  ***(N=238)*** | |  | | | ***Study time-point I&II***  ***(N=172)*** | | |  | | ***P*** | |  |
|  | |  |  |  |  |  | |  | | | |  | |
| **Variables** | | **Mean±Standard Deviation** | | | | | | | | | | | |
| ***CDDQ* *subscales*** | |  | |  |  | |  |  | |  | | |  |
| - Tension and discomfort | | 1.81±0.73 | |  |  | | 1.80±0.79 |  | | *0.854* | | |  |
| - Embarrassment | | 1.79±1.04 | |  |  | | 1.76±1.09 |  | | *0.739* | | |  |
| - Concern about sexual and reproductive consequences | | 1.41±0.83 | |  |  | | 1.32±0.88 |  | | *0.259* | | |  |
| - Concern about health consequences | | 2.44±1.04 | |  |  | | 2.40±1.12 |  | | *0.705* | | |  |

CDDQ: the Cervical Dysplasia Distress Questionnaire. *P (probability,* t-test).

There was no significant difference between the subjects with a positive Papanicolaou test who participated in the first (*Study time-point I*) and second (*Study time-point II*) time points of the study in relation to the psychosocial status of the subjects in cervical cancer screening according to the POSM scale (Table 13).

| **Table 13. Psychosocial status of respondents in cervical cancer screening according to the POSM scale** | | | | | | | | | | | | | | |
| --- | --- | --- | --- | --- | --- | --- | --- | --- | --- | --- | --- | --- | --- | --- |
|  | ***Study time-point I***  ***(N=238)*** | | |  | | | ***Study time-point I&II***  ***(N=172)*** | | |  | | ***P*** | |  |
|  | |  | |  |  |  |  | |  | | | |  | |
| **Variables** | | **Mean±Standard Deviation** | | | | | | | | | | | | |
| ***POSM* *subscales*** | |  | | |  |  | |  |  | |  | | |  |
| - Worry | | 39.09±18.55 | | |  |  | | 38.52±19.89 |  | | *0.764* | | |  |
| - Satisfaction with information/support | | | 40.22±17.64 | |  |  | | 39.47±18.74 |  | | *0.683* | | |  |

POSM: Process and Outcome Specific Measure. *P (probability,* t-test).
